# Editorial: A global perspective on current research in physiology education

**DOI:** 10.1016/j.crphys.2026.100188

**Published:** 2026-08-01

**Authors:** Sara Namvar, David Greensmith

**Affiliations:** Biomedical Research and Innovation Centre, The University of Salford, Salford, M5 4WT, United Kingdom

In the challenging landscape of education, there exists a constant need to innovatively adapt our practices to meet the complex needs of learners. Only by doing this can we make higher education truly inclusive, accessible and effective for all (Namvar et al., 2021a, 2021b, 2023). This special issue was conceived to capture this evolving dynamic and to showcase evidence-based innovations that are redefining how curricula are taught and experienced.

The articles contained within feature progressive pedagogy that spans the entire educational pipeline—from secondary school outreach to graduate employability. Together, they demonstrate that effective teaching requires the same rigorous evaluation as the physiological systems we study. While the context here is physiology, the principles covered are largely universal so ought to be useful to educators across a broad range of academic disciplines.

## Widening participation and improving accessibility

Before students can succeed at University, we must understand the barriers that limit access to degree programmes. Here, Greensmith and Greensmith (2025) address the challenge of socioeconomic background and demonstrate that effective high school outreach programmes can improve awareness of STEM careers and the pathways required to achieve them. For those who do reach university, educators must recognise that conceptual understanding develops individually. Towards this, Kuang et al. (2025) unravel the causes of student misunderstanding using tonicity as an example.

## Enhanced learning through innovation

Introduction of innovative delivery modalities can enhance inclusive core learning. Morgan et al. (2025) and Bagley et al. (2025) explore how gamification through digital escape rooms and storytelling can improve analytical and problem solving skills by reducing associated apprehension among students. In other work, Amar et al. (2025) leverage immersive simulations to improve applied clinical reasoning thus boost confidence.

Graduate employability relies on the ability to translate conceptual understanding into effective application. This is heavily dependent on the co-development the wider academic skills that students frequently find challenging. Swainson et al. (2025) address this by creating an interactive, self-guided tutorial designed to facilitate construction of logical arguments and subsequent peer review while Armstrong et al. (2025) present a science communication-focused summer project that boosts students' transferable skills.

Building on the potential of digital connectivity, Jones et al. (2025) demonstrate how Collaborative Online International Learning (COIL) can transcend geographical boundaries to create international learning communities, enhance scientific communication and foster cross-cultural competencies. Meanwhile, Alagbonsi et al. (2026) evaluate wider stakeholder perceptions surrounding online delivery.

## The integration of artificial intelligence

The advent of generative artificial intelligence (AI) presents both challenges and unparalleled opportunities for higher education. Here, Hardy (2025) critically examines the use of Large Language Models (LLMs) to actively teach academic writing and critical appraisal skills. The administrative and pedagogical implications of AI are further investigated by Reid et al. (2025), who evaluate perspectives on AI-generated multiple-choice questions in assessments.

## Looking forward

Together, these contributions provide a unifying theme: physiology education thrives when we prioritise equity, embrace active learning, leverage emerging technologies responsibly, and continuously reflect on our practice. We extend our sincere gratitude to all contributors. It is our hope that this special issue not only provides practical frameworks for today’s educators but also inspires the next wave of pedagogical research and innovation.

## References

[bib1] Alagbonsi A.I., Gbadamosi M.A., Nemerimana M., Olubiyi M.V., Onaadepo O., Ime A.U., Feyitimi A.-R.A., Mungai R.W., Rutagarama F., Zambrano D.M., Omeiza N.A., Elias S.O. (2026). How effective is online teaching of physiology modules? Perception of Rwandan health sciences students. Curr. Res. Physiol..

[bib2] Amar K., Jones M., Connell N., Mayo D., Forde L., Sayan B.S., Lorente-Pons A., Magwaza R.N., Giachino A., Nirmalan N., Namvar S. (2025). Enhancing career development for biomedical sciences students: leveraging simulations to support patient-facing careers. Curr. Res. Physiol..

[bib3] Armstrong V.L., Lawry B.M., Stevenson-Cocks H.J. (2025). A science communication-focused summer project boosts first year bioscience students’ skill gains and supports placement year uptake. Curr. Res. Physiol..

[bib4] Bagley L., Willsone A., Kime A. (2025). The story so far………- current opinion in the use and applications of interactive storytelling in physiology and clinical education. Curr. Res. Physiol..

[bib5] Greensmith C., Greensmith D. (2025). You don't know what you don't know; using high school outreach to improve awareness of bioscience-based careers and higher education. Curr. Res. Physiol..

[bib6] Hardy M. (2025). Academic writing and critical appraisal in physiology education: discerning the benefits of using large language models as knowledge receivers or knowledge providers. Curr. Res. Physiol..

[bib7] Jones M.A., Miklavc P., Stewart M. (2025). Enhancing laboratory education through collaborative online international learning: a case study between USA and UK students. Curr. Res. Physiol..

[bib8] Kuang S.Y., Yang X., Li X. (2025). Unravelling tonicity: causes of confusion and pathways to clarity. Curr. Res. Physiol..

[bib9] Morgan N., Yaqoob M., Jones M.A. (2025). Using digital escape rooms to enhance data analysis skills and student experience in a higher education human physiology module. Curr. Res. Physiol..

[bib10] Namvar S., Greensmith D., Nirmalan N. (2021). Co-creation and empowerment: pathways to better student engagement. Times Higher Education.

[bib11] Namvar S., Greensmith D., Nirmalan N. (2021).

[bib12] Namvar S., Pinnington A., Courchene P., Greensmith D., Nirmalan N. (2023). Contemporary Practices and Initiatives in Employability - an Advance HE Case Study Compendium.

[bib13] Reid M., French M., Andreopoulos S., Wong C., Kee N. (2025). AI-generated multiple-choice questions in health science education: stakeholder perspectives and implementation considerations. Curr. Res. Physiol..

[bib14] Swainson A., Mason M.J., MacMillan F.M. (2025). An interactive, self-guided tutorial on scientific writing for first year physiology students. Curr. Res. Physiol..

